# Biochar-Assisted Agriculture: From Healthy Soil to Healthy Plants

**DOI:** 10.3390/plants14213273

**Published:** 2025-10-27

**Authors:** Cheng Liu, Chao Wang, Shijie Shang, Jingyu Ma, Shengdao Shan, Qian Yue, Lianqing Li, Genxing Pan

**Affiliations:** 1 Key Laboratory of Recycling and Eco-treatment of Waste Biomass of Zhejiang Province, School of Environment and Natural Resources, Zhejiang University of Science and Technology, Hangzhou 310023, China; 2 Institute of Agricultural Resources and Environment, Jiangsu Academy of Agricultural Sciences, Nanjing 210042, China; 3 Institute of Resource, Ecosystem and Environment of Agriculture, Nanjing Agricultural University, Nanjing 210095, China

**Keywords:** biochar, soil health, root growth, crop yield, plant resistance

## Abstract

Land application of biochar appears to be the most promising tool for managing soil and plant health in agriculture for food production. Biochar induces plant resistance and root growth, deactivates fungitoxic compounds, supports better habituation of beneficial microorganisms, and alters soil properties to facilitate moisture and nutrient availability. This review assimilates lessons from the authors’ experience with biochar application in agriculture, in addition to the previous literature, to elucidate the role of biochar in crop production, from soil health to plant health (root growth, disease control, yield, and quality), and its link to food health. This review provides bottom–up evidence for developing biochar-assisted agriculture in the context of ONE Health applied to soil–plant–food, contributing to achieving the United Nations Sustainable Development Goals (UNSDGs).

## 1. Introduction

Soils are fundamental to Earth’s sustainability, playing a central role in driving biogeochemical nutrient cycles and delivering critical ecosystem services. These include carbon sequestration, water retention and purification, the immobilization of toxic metals and organic pollutants, biodiversity conservation, and landscape stabilization [1,2]. Soils serve as essential physical habitats that sustain underground biodiversity and condition the physical environment, thereby enhancing landscape beautification. Consequently, healthy soils are integral to the One Health of all global life forms [1]. Over the past two decades, global soils have been at risk due to extensive soil degradation, climate change, biodiversity loss, and environmental pollution [3]. Nature-based solutions are essential to address the multifaceted challenges of achieving the UNSDGs by 2030 and beyond. Among these, the safeguarding and sustainable management of Earth’s soils are fundamental to securing global agriculture and food supplies [4].

Biochar is derived from the pyrolysis of biomass residue under limited oxygen. Research attention on biochar has increased significantly since 2000, following early findings on Terra Preta soils that highlighted biochar’s ability to improve soil properties, raise agricultural productivity, and help mitigate climate change [5,6]. It is produced from feedstocks including wood residues, straw, manures, sewage sludge, and food waste at high treatment temperatures (HTTs) between about 350 °C and 750 °C. Biochar properties are highly diverse and governed primarily by feedstock selection, HTTs, residence time, and any treatments before or after pyrolysis [7]. Ippolito et al. [8] concluded that wood-based biochars have the highest surface area, straw-based have the highest CEC, and manure-based have the highest N and P levels, and that HTTs over 500 °C yield more persistent biochars with increased ash content and pH.

**Figure 1:**
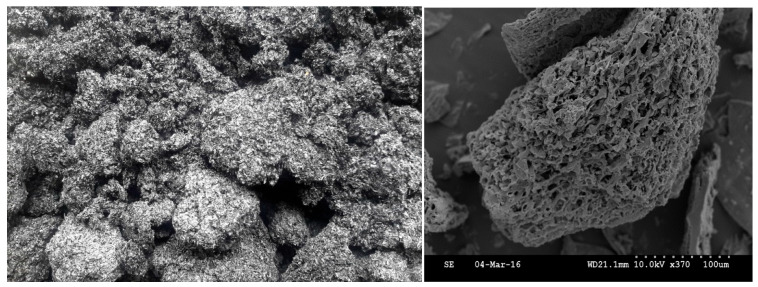
The spongy biochar mass (left) from agricultural biomass (cattle manure) pyrolyzed at 550 °C and its porous structure (right), as shown by scanning electron microscopy according to Lu et al. [9] (photo by Dr. Pan in 2016).

Converting organic waste from agriculture into biochar and amending it into agricultural soils has received great attention over the last 15 years (Figure 1). Biochar production and soil amendment are widely recognized as a promising strategy for managing biomass waste and enhancing soil health on a global scale. This approach draws inspiration from the historical and scientific rediscovery of Terra Preta, a highly fertile dark earth found in the Amazon Basin of South America. Soils in the Amazon region are highly impoverished due to intense weathering under warm and humid climatic conditions [5]. They are very acidic and have a low cation exchange capacity to retain nutrients. In addition, the soil organic matter content is extremely low due to the rapid decomposition of organic matter in the warm and wet climate. All of these issues challenge the sustainable management of agriculture in most tropical regions.

In the first decade of the twenty-first century, numerous studies were published advocating the conversion of biomass waste into biochar via pyrolysis and its incorporation into soils to sequester carbon and mitigate climate change. Lehmann [10] estimated that pyrolyzing forest residues could reduce carbon release by 25% and sequester approximately 10% of the United States’ annual fossil fuel emissions. Compared with burning—which retains only about 3% of carbon—or biological decomposition, converting biomass carbon into biochar enables approximately 50% of the original carbon to be stored. Furthermore, Lehmann et al. [6] suggested that replacing slash-and-burn agriculture with slash-and-char techniques could offset up to 12% of global anthropogenic carbon emissions each year via soil sequestration. Beyond its role in carbon storage and climate mitigation, biochar soil amendment was also recognized for its potential to enhance soil productivity and increase crop yields [10].

For the last decade, more studies focused on the impact of biochar on plant root growth [11], plant diseases, including grain diseases and cash crop diseases [12], and plant health and food quality [13,14,15]. Nevertheless, a broad range of field experiments have been conducted to explore the potentialto use biochar for soil and plant health improvement before large-scale biochar implementation. Based on individual case studies, this review gives a general conclusion on biochar’s effect on soil fertility, crop yield, crop root growth, plant disease suppression, and crop quality (nutritional quality and functional quality). We hope that this review will enhance the understanding and awareness of biochar application in the context of One Health.

## 2. Biochar for Plant Production: Multifaceted Functionality

### 2.1. Soil Fertility Improvement

Soil amendment biochar has been well adopted as a promising strategy and practical tool to boost soil fertility in large-scale agricultural production. This points mainly to the improvement in soil biophysical architecture, leading to higher soil porosity and consequently to higher water and carbon retention, higher soil adsorption and exchange capacity induced by the porous structure and large surface area, and a higher pH. Therefore, biochar amendment can mitigate soil compaction by improving soil aggregation by 12–16% [16]. Soil bulk density has been shown to decrease by 7.6% to 29%, while soil porosity has increased by 7% to 59% across multiple studies [16,17,18,19]. The positive effect of biochar on soil aggregation was more pronounced in loam-textured, neutral, and acidic soils [16]. Biochar derived from wood residues pyrolyzed at high temperatures (>600 °C) exhibited a stronger enhancing effect on soil aggregation [20].

High-temperature-produced biochar has a lower atomic ratio of O/C and is inherently hydrophobic [21]. While biochar promotes sequestration of fresh SOM in soil aggregates, its hydrophobicity considerably lessens the dispersion of soil aggregates [22]. These structural improvements contributed to better water retention in the soil. In biochar-amended soils, the available water content was generally higher compared with untreated soils. Specifically, the soil water-holding capacity increased by 10–29% following biochar application in previous studies [16,19,23]. Biochar was more effective in increasing the available water content in coarse-textured soils than in fine-textured ones, as evidenced by a meta-analysis showing increases of 31.4% versus 13.6%. In coarse-textured soil, biochar application rates of 30–70 t ha^−1^ increased the available water content by 23.5% compared to a 30 t ha^−1^ application rate [23]. In fine-textured soil, comparatively, the changes in available water content was smaller (by 16.4% at 30 t ha^-1^) but insignificant at 71–200 t ha^−1^.

Biochar significantly influences soil nutrient status, availability, and biogeochemical cycling processes [24]. Feedstocks such as animal manure and crop residues used for biochar production are inherently rich in nutrients. During pyrolysis, a substantial portion of these nutrients, including phosphorus, potassium, calcium, magnesium, and silicon, is retained as ash in the resulting biochar. Upon incorporation into the soil, these nutrients become available for plant uptake. A meta-analysis by Biederman and Harpole [25] concluded that biochar amendment significantly enhances the concentrations of phosphorus (P), potassium (K), nitrogen (N), and organic carbon (C) in soil. Furthermore, biochar has been shown to increase soil pH by up to 1.2 units in highly acidic environments [26]. Numerous studies indicate that biochar alters the dynamics and cycling of soil nutrients, thereby either enhancing or reducing their availability to plants. Nitrogen and phosphorus are among the most frequently affected nutrients. For instance, Gao et al. [27] and Tesfaye et al. [28] reported that biochar application increased soil available phosphorus content by 45–65% on average across individual studies, while plant phosphorus uptake rose by an average of 55%. Nevertheless, the highest effect was observed in strongly acidic and fine-textured soil or in soils with an initial low P availability.

Biochar amendment in agricultural soils decreases the concentration of inorganic N, for example, the concentration of NO_3_^−^ or NH_4_^+^ decreased by 10–12% [27,29]. Such reduction could be atrributable to biochar N immobilization and limited microbial nitrificiation due to microbial community shift following biochar amendment. Despite, biochar addition increases N fertilizer use efficiency as it decreases N loss from soil [30]. Biochar captures N more slowly, and it is then slowly released and taken up by plants. Biochar could decrease N loss via nitrate leaching and N_2_O emission during nitrification and denitrification, positively affecting NO_3_^−^ and NH_4_^+^ reduction [31]. Albeit, ammonium volatilization may be unlikely impacted [32]. In addition, micro-nutrients are supplemented with biochar after incorporation . Their availability and cycling are altered, but this needs further study. For example, Major et al. [33] reported a long-lasting yield increase effect of one-time biochar application, which they attributed to the increased availability of soil calcium and magnesium. In rice paddies, Liu et al. [34] found that biochar significantly increased the plant available Si pool, and rice shoot Si uptake increased by up to 58%.

### 2.2. Plant Rooting Promotion

**Figure 2:**
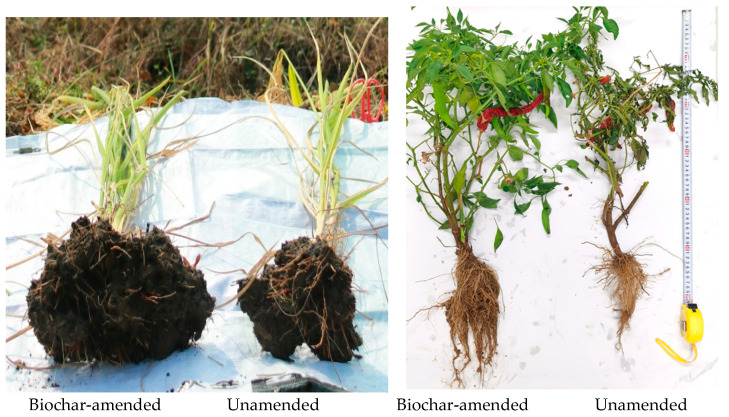
The root volume of an Indica rice hill (left) and a plant of Capsicum chili pepper (right) in a biochar-amended field compared with an unamended field (photo by Dr. Pan in 2015 and 2020, respectively).

A global meta-analysis by Xiang et al. [11] revealed biochar application’s great effect on increasing root biomass by 32%, root volume by 29%, surface area by 39%, root length by 52%, the number of root tips by 17%, and root diameter by 9.9%. Significant increases in volume and root length have often been observed in field studies using biochar soil amendment for cereals and vegetable crops (see Figure 2 for an example in rice and chili pepper). In a pot experiment [35], the number of fine roots (diameter <0.2 mm) increased with biochar addition. This result suggests that biochar application benefits root morphological development, alleviating plant nutrient and water deficiency rather than maximizing biomass accumulation. We previously applied biochar to a maize field and found that it increased the biomass of corn roots and improved the root morphology of maize, thereby enhancing the yield [36]. In an experiment on biochar’s effect on the root growth of perennial crops, we found that ginseng root biomass increased by 25–27% at 20 t ha^−1^ of biochar made of lignocellulose biomass compared with conventional manure compost [13]. Moreover, biochar can increase the number and weight of root nodules in leguminous crops, such as soybeans and peanuts, thereby promoting nitrogen fixation in peanuts and enhancing the yield by ca. 10% [14,15].

These findings may be attributed to differences in root strategies between the two plant life forms. Compared with perennial plants, which typically develop root systems that maximize nutrient conservation, annual plants generally enhance root growth to facilitate greater nutrient acquisition [11]. Therefore, annual plants are likely to exhibit more efficient root growth compared with perennial plants, owing to the improved soil nutrient conditions resulting from biochar amendment. However, due to the limited number of studies and small sample sizes, it is not possible to draw definitive conclusions regarding the influence of biochar on root traits across different plant life forms. The stronger root biomass response observed in legumes relative to non-legumes may be attributed to enhanced nodulation and more efficient nitrogen fixation in biochar-treated leguminous plants [14,37]. In particular, biochar-mediated nutrient enrichment positively influenced diazotrophic gene abundance while also playing a significant role in structuring these communities [37].

### 2.3. Plant Productivity Boost

Globally, over 200 field and pot experiments have extensively investigated the effects of biochar amendment on plant growth, particularly crop production (Table 1). These meta-analyses demonstrated that amending agricultural soils with biochar can significantly enhance crop productivity, increasing both shoot biomass and grain yield. The percentage increase ranged from 0% to 32%, with an average of 15% across studies. This considerable variation can be attributed to differences in the target variables used. Early studies often employed the broad term “crop productivity” to encompass as many case studies as possible. This metric referred to grain yield in cereals, biological yield in vegetables, fruit yield in fruit-bearing plants, and shoot or root biomass in most other plants. As the number of case studies rapidly grew, later research shifted its focus specifically to grain yield in field-based investigations.

A number of biotic and abiotic conditions regulate the response of crop productivity to biochar amendment, including crop type, soil acidity and texture, biochar feedstock, climate, and the application rate of N fertilizers. Generally, tuber/root vegetables have the highest yield response to biochar amendment, followed by rapeseed, other vegetables, and the main crops of maize, wheat, and rice [48]. Maize has a relatively higher grain yield response than wheat and rice, and rice has the lowest response [39,41,44,46,48]. The percent increases for maize, wheat, and rice are 14.3%, 8.0%, and 3.4%, respectively, according to the latest study [39]. Legume crops’ response to biochar amendment varied across studies. Some studies observed a significantly higher yield response for legumes compared to other main crops [39,44,46], while others found that biochar had no/little effect on legumes [41,48]. Nevertheless, biochar is often recommended for use in dryland crops, especially for soybean, maize, and wheat. Soil acidity and texture are two key factors that influence crop yield response to biochar in target soils enriched with biochar amendment. When adding biochar to acidic or coarse-textured soils, a greater yield response is expected than when adding it to neutral/alkaline or fine- and medium-textured soils [18,41,42,44,45,46]. However, in rice paddies, a greater rice yield and N use efficiency were observed in alkaline or fine-textured soil than in acidic or coarse-textured soil [40].

The properties of biochar are influenced by multiple factors, such as feedstock type, pyrolysis technology, and temperature. Among these, the feedstock source plays a major role in determining the crop yield response to biochar amendment. In general, biochar derived from livestock manure tends to exhibit a higher potential for increasing crop yields compared with those from crop straw or wood residues [40,41,46,48]. However, the findings regarding the effectiveness of different plant-based biochars are inconsistent. For instance, Liu et al. [46] reported that wood residue biochar had a stronger yield-increasing effect than straw-derived biochar, while other studies found the opposite [41,44,46]. In contrast, Bai et al. [48] and Liu et al. [40] observed comparable effects between the two. Additionally, biochar produced at pyrolysis temperatures between 350 °C and 450 °C exhibits a greater yield-enhancing potential than biochar generated at other temperatures. Jeffery et al. [45] highlighted the role of climate in moderating crop yield responses to biochar, noting that biochar amendments tend to increase yields in tropical regions but show limited effects in temperate regions. This discrepancy may be attributed to the typically acidic and less fertile soils found in tropical areas, where biochar may help ameliorate soil constraints more effectively.

### 2.4. Plant Biodefense Manipulation

**Figure 3:**
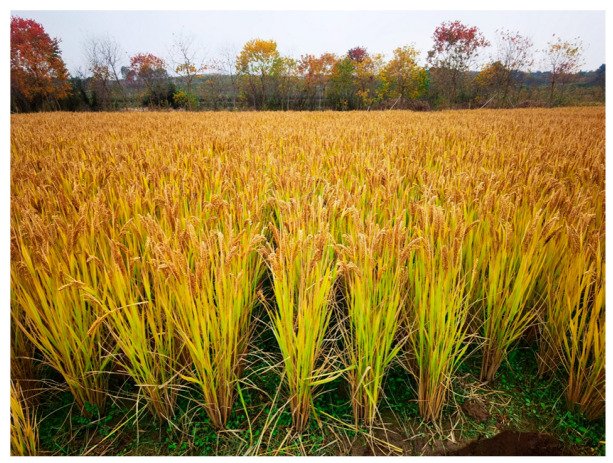
Japonica rice at harvest in a sandy loam paddy amended with rice husk biochar at 15 t ha^−1^ (photo by Dr. Genxing Pan in 2020).

Biochar’s direct and indirect changes in the rhizosphere soil, host plant, pathogen, and rhizosphere microbiome can have multifactorial impacts on both plant development and disease progress. Due to biochar’s specific chemical properties, abundant nutrients, and porous structure, biochar can recruit microbes and reshape the structure of the microbial community in soils [49]. An early report on biochar application for plant disease suppression showed that biochar derived from citrus wood induced systemic resistance to *Botrytis* *cinerea* and *Leveillula* *taurica* in both pepper and tomato [50]. In the following 10 years, more studies focused on biochar’s impact on plant diseases, including grain diseases and cash crop diseases [12]. Biochar-induced soil health improvement contributed to the increased microbial diversity and activity in the biochar-amended rhizosphere, a potential mechanism of biochar-induced system resistance [9] (Figure 3 shows the experimental site) and the suppression of soil-borne plant diseases [51,52]. These effects were potentially caused by reductions in pathogen colonization in the soil and enhancements in the growth of beneficial microorganisms [13,52]. A recent global meta-analysis by Yang et al. [12] demonstrated that biochar amendment dramatically reduced disease severity by 47.5% on average.

(1) The liming effect: Biochar is typically alkaline and has been widely reported to elevate soil pH. As pH plays a critical role in shaping microbial community development, diversity, structure, and pathogen virulence, such alterations can have profound ecological implications. Given that many soil pathogens are adapted to narrow pH ranges [53], biochar-mediated shifts in rhizosphere pH may significantly influence pathogen survival and activity.
(2) The supply of organic compounds varies with the type of biochar feedstock, as different raw materials lead to differences in elemental composition and ash content [54]. These variations affect the concentrations of active components—such as soluble organic compounds—in the resulting biochars, which likely explains their divergent effectiveness in suppressing plant diseases [55]. Integrated global database analysis has also confirmed that biochar can suppress the occurrence of soil-borne diseases and plant diseases [12].
(3) Biochar can deactivate toxic compounds released by roots through its strong adsorption capacity, attributed to its high surface area and porous structure [52]. For example, Jaiswa et al. [55] demonstrated that wood chip and greenhouse pepper waste biochar (350 °C and 600 °C) can adsorb and deactivate cell wall-degrading enzymes and toxic metabolites produced by the pathogen *F. oxysporum f.* sp. *radicis-lycopersici*, thereby protecting tomatoes from soil-borne pathogens. Gu et al. [52] found that the application of pine biochar can directly or indirectly adsorb root exudates (thereby reducing the chemotactic ability of pathogens) to attract pathogens, while significantly impairing their motility and colonization, ultimately reducing the pathogenicity of pathogens toward tomatoes. Growing evidence supports biochar’s role in immobilizing allelochemicals derived from root exudates [52] and suppressing soil-borne pathogens [51]. In our study on biochar amendment in replanted ginseng (*Panax ginseng*), biochar markedly reduced the accumulation of root-derived phenolic allelochemicals, thereby inhibiting soil-borne pathogenic fungi, while simultaneously enhancing microbial diversity and network complexity [13].
(4) Soil microbial manipulation is a critical approach to addressing the challenges posed by continuous cropping, which adversely affects soil health and promotes soil-borne diseases. These conditions further disrupt soil properties, alter microbial community structure, and lead to pathogen accumulation in the rhizosphere [56,57]. Biochar amendment has been shown to promote beneficial microorganisms while reducing the abundance and pathogenicity of pathotrophic fungi. Notably, maize biochar outperforms wood biochar in enhancing the abundance of arbuscular mycorrhizal fungi (AMF) and beneficial bacteria [13]. Additionally, biochar application increases the complexity of microbial co-occurrence networks, particularly within fungal communities [13,57]. Consequently, the core microbial networks exhibit enhanced resistance even as pathogenic fungi proliferate in biochar-amended soils. Biochar also promotes the enrichment of plant-growth-promoting rhizobacteria (PGPR) in the rhizosphere via host-mediated recruitment. For instance, Jin et al. [58] demonstrated that biochar stimulates tomato roots to assemble a protective bacterial community that confers resistance to *Fusarium* wilt.
(5) Biochar-induced plant resistance: Biochar soil amendment can directly influence the physiological status of plants, particularly by modifying root exudation, which facilitates the recruitment of plant-growth-promoting rhizobacteria. Previous studies have indicated that biochar exerts direct effects on plant growth and physiological processes [59,60]. Moreover, biochar shows considerable potential in activating immunity-related gene expression. Transcriptomic analyses in tomato have revealed that biochar primes defense-related pathways, upregulating genes and hormones associated with plant immunity and development, including jasmonic acid, brassinosteroids, cytokinins, and auxin, and the synthesis of flavonoids, phenylpropanoids, and cell wall components [61]. In Kong et al.’s study [62], exogenous application of nanoscale biochar was shown to enhance plant defense responses and confer resistance against the pathogen Phytophthora nicotianae. These findings suggest that biochar, when applied at levels that optimally stimulate plant immunity, could serve as an effective plant protection agent in future agricultural practices.

### 2.5. Food Quality Enhancement

Biochar improves soil quality, enhances root growth, and increases crop yield; however, in recent years, a growing number of studies have found that biochar can also enhance crop quality. A meta-analysis by Lei et al. [63] revealed that biochar application significantly increased total soluble solids (4.28%) and vitamin C content (6.77%). The study further noted that these benefits were more significant at higher application rates. These findings indicate that biochar contributes to health from the perspective of both plant development and human nutrition.

Moreover, the study demonstrated that biochar amendment could alter kernel composition and improve oilseed quality in peanuts [15,16]. Despite relatively minor and inconsistent changes in peanut yield across the different amendment treatments, all biochar amendments led to substantial improvements (approximately 10%) in kernel composition and oilseed quality compared with traditional organic fertilizer [15,16]. In previous studies, peanut oilseed composition and quality, particularly fatty acid abundance, have been reported to be minimally affected by field conditions, such as climate change simulations [64] and irrigation performance evaluations [65]. Biochar amendments induced slight but significant increases in the crude protein content of peanut kernels, which correlated with the ratio of oleic acid to linoleic acid (O/L ratio) and the ratio of oleic acid to palmitic and stearic acid (O/(P + S) ratio). The ratio of oleic acid to linoleic acid, which is crucial for human health [66,67], was altered by up to 30% with maize straw biochar compared with the control, meeting the Chinese standard for high-oleic oilseed [68]. According to Seleiman et al. [69], sunflower oil and oleic acid contents decreased by 18% and 26%, respectively, under severe water stress compared with normal conditions. This aligns with the findings of Khan et al. [70], who reported 10% and 12% increases in oil and oleic acid contents in rapeseed plants treated with a combination of biochar amendments under severe water stress, respectively. In addition, biochar amendments can strongly affect the biosynthesis of secondary metabolites [68,69,70,71]. For instance, rapeseed treated with wood waste biochar at 10 t ha^−1^ exhibited an increase in the ratio of polyunsaturated to saturated fatty acids [69]. According to Gómez [72], the fatty acid content of artichokes was increased by the application of 13 t ha^−1^ biochar produced from a mixture of untreated log charcoal, vinasse, and sugarcane molasses, an effect attributed mainly to palmitic acid.

Another study found that biochar increased the health of ginseng root quality, determined by its ginsenosides, and reduced residual pesticides [13]. These findings suggest that biochar could significantly improve the quality of crops even under sick soil conditions. Biochar technology may boost high-quality food production in farmlands.

## 3. Potential Toxicity When Applied in Soil

Scaling up any technology must be predicated on addressing environmental regulations and considerations to prevent adverse ecological and health impacts. The potential presence of hazardous compounds in biochar raises concerns regarding its risks to environmental health. The primary origins of contamination can be categorized as follows: (1) Feedstock source: Certain materials, including industrial wastes and chemically treated biomass, inherently possess a higher likelihood of containing contaminants or their precursors, making their utilization for biochar a subject of concern [73,74,75]. (2) Pyrolysis temperature: Lower temperatures or excessively high temperatures are known to produce toxic compounds, notably persistent aromatic structures [75,76]. Current concerns center on the leaching and environmental fate of aromatic organic compounds and heavy metals from biochar, though the extent of these effects remains inadequately quantified.

In this context, a critical priority is to assess the potential effects of biochar on recipient environments following soil application. Biochar does contain various amounts of potentially toxic compounds such as PAHs, raising concerns for plant health and food security [77]. However, biochar from primary biomass such as crop residue, including straw from maize, rice, or wood, excluding sludge or manure, contains very low concentrations of PAHs within the limit of 6 mg kg^−1^ set by the IBI [78] or 4 mg kg^−1^ by the EBC [79]. According to Yao et al. [80], the bioavailability of PAHs in biochar is indeed very low, at the level of several ng/kg, leading to plant uptake and thus food risk. We should pay attention to the potential soil contamination of PAHs via land application of biochar, but PAHs in SOM-rich soil generally bind to the complex matrix of soil organic matter, which poses a negligible risk for adsorption or uptake by soil fauna [81]. This is particularly true in field conditions [82]. In our field application, we used biochar mostly from rice and maize biomass pyrolyzed under oxygen-depleted conditions at 450–500 °C, containing low contents of total PAHs. Toxic accumulation in plant edible parts was not found nor were adverse effects on plant/seedlings observed. In contrast, using biochar improved plant health and thus improved food quality and nutrition. Lyu et al. [83] reported that biochar produced at temperatures >400 °C generally exhibits reduced toxicity, making it more suitable for soil amendment. This is attributed to the stabilization of potentially toxic elements, such as heavy metals, under elevated pyrolysis temperatures.

Recent research has focused on developing methods to mitigate the toxicity of biochar. For instance, Asad et al. [84] successfully employed a co-plasma processing technique to produce potassium- and sulfur-rich biochar from banana peduncle biomass, a method that also avoids reactor-derived contamination. This innovative approach addresses several limitations inherent in conventional pyrolysis, including restricted heating rates, limited peak temperatures, and prolonged production time [85]. Despite its advantages, the application of this method for biochar production remains scarcely reported. The authors demonstrated that the resulting biochar exhibited a reduced leachable fraction of fluoride and heavy metals, yielding a nutrient-rich product with lower toxicity. Such advancements underscore the importance of integrating sustainability measures into biochar production.

## 4. Challenges and Prospects

While biochar offers significant potential for soil amendment, concerns remain regarding its behavior and fate in the environment. A thorough understanding of its long-term impact is crucial for preventing adverse ecological effects, such as ecotoxicity, and for ensuring the sustainability of its application. To achieve this, there is an urgent need to develop standardized analytical methods for tracking biochar in the environment. Comprehensive studies are also needed on biochar’s long-term influence on soil characteristics, its mobility which should be precisely assessed.

Subsequent studies could focus on the role of biochar as a key component in the transition toward sustainable agriculture, addressing challenges related to climate change, environmental protection, and food security. The function of biochar in fertilizers extends beyond adsorption and retention. Exploring the potential benefits of combining biochar with other soil amendments or fertilizers may enhance its capacity to address specific soil deficiencies or problems. This could include investigating formulations that optimize nutrient retention, pH balance, and microbial activity.

It is crucial to conduct thorough studies of the environmental implications related to large-scale biochar production and use. This involves assessing carbon footprint, energy use, and potential emissions to verify that biochar usage aligns with sustainability objectives.

Most studies are conducted over short-term periods, and uncertainty remains about whether biochar’s effectiveness in crop growth persists after its prolonged incorporation in the field. Performing extended field research to evaluate the lasting impacts of biochar use on soil characteristics and production would be beneficial. Some knowledge gaps regarding biochar application for healthy plants are detailed as follows:

(1) To what extent are plant processes affected by soil changes (abiotic versus biotic)?
(2) How do plant roots respond to biochar material input versus biochar (habituating versus signaling)?
(3) What mediates the interplay between plant growth, resistance, and biosynthesis following biochar soil application?
(4) What will the legacy of the soil–plant system be following biochar application?

Enhancing awareness and understanding of biochar among farmers, land managers, and policymakers is crucial to promoting its broad and sustainable adoption. Outreach efforts should prioritize offering practical guidance on application techniques, appropriate dosages, and context-specific recommendations tailored to different soil types and farming systems. It is also essential to conduct cost–benefit analyses and assess both the internal and external impacts of biochar application in specific regions. The number of studies on the economic aspects of biochar produced from various feedstocks under different pyrolysis conditions remains limited in the literature. This scarcity poses challenges for decision-makers in selecting the most cost-effective technology among available alternatives. Therefore, it is recommended that future reports on the preparation and application of biochar for soil use include a dedicated section on economic evaluation. Additionally, there is a need for social science research to understand public perception regarding biochar use and to identify strategies for overcoming barriers that may restrict its broader adoption in soil amendment. Furthermore, conducting a meta-analysis of existing biochar studies would help integrate and validate findings across diverse research efforts. Ultimately, initiatives aimed at encouraging farmers to incorporate biochar into their soil management practices are unlikely to succeed without adequately addressing its economic feasibility.

## Figures and Tables

**Table 1:** The meta-analyses on biochar’s effect on crop production published since 2011.

Authors and Year	Change in Crop Productivity	Observation	Experiment
Amirahmadi et al. (2025) [38]	Yield: 16.2%	1166	Field
Singh et al. (2022) [18]	Crop yield: 32%	110	Pot and field
Zhang et al. (2022 ) [39]	Crop productivity: 13%	691	Field
Liu et al. (2022) [40]	Yield: 10.7%	378	Pot and field
Farhangi-Abriz et al. (2021) [41]	Grain yield: maize—28%; wheat—13%	296	Field
Dai et al. (2020) [42]	Plant productivity: 16%	1254	Pot and field
Liu et al. (2019) [43]	Yield: 15.4%	605	Field
Ye et al. (2019) [44]	Yield: 9.9%	232	Field
Jeffery et al. (2017) [45]	Yield: 13%	1125	Pot and field
Biederman and Harpole (2013) [25]	Biomass*_ag_*: 29.7%; biomass*_bg_*: 39.9%; yield: 18.7%	317	Pot and field
Liu et al. (2013) [46]	Biomass: 12.5%; yield: 8.4%	880	Pot and field
Jeffery et al. (2011) [47]	Biomass*_ag_* and grain yield: 10%	177	Pot and field

Note: Biomass_ag_ and biomass_bg_ represent biomass aboveground and belowground, respectively.

## Data Availability

No experimental data were created in this study. Data sharing does not apply to this article.
